# A new Newton-like method for solving nonlinear equations

**DOI:** 10.1186/s40064-016-2909-7

**Published:** 2016-08-05

**Authors:** B. Saheya, Guo-qing Chen, Yun-kang Sui, Cai-ying Wu

**Affiliations:** 1 College of Mathematics Science, Inner Mongolia University, Hohhot, 010021 China; 2 College of Mathematical Science, Inner Mongolia Normal University, Hohhot, 010022 China; 3 College of Mechanical Engineering and Applied Electronics Technology, Beijing University of Technology, Beijing, 100124 China

**Keywords:** Rational approximate function, Improved Newton’s method, Local convergence, 90C25, 90C30

## Abstract

This paper presents an iterative scheme for solving nonline ar equations. We establish a new rational approximation model with linear numerator and denominator which has generalizes the local linear model. We then employ the new approximation for nonlinear equations and propose an improved Newton’s method to solve it. The new method revises the Jacobian matrix by a rank one matrix each iteration and obtains the quadratic convergence property. The numerical performance and comparison show that the proposed method is efficient.

## Background

We consider the system of nonlinear equations

1 $$\begin{aligned} F(x)=0, \end{aligned}$$

where $F:{\mathbb {R}}^n\rightarrow {\mathbb {R}}^m$ is a continuously differentiable function. All practical algorithms for solving (1) are iterative. Newton’s method is the most widely used method in applications (see Traub 1964; Ortega and Rheinboldt 1970; Dennis and Schnabel 1993; Kelley 2003; Petković et al. 2013a).

The linearization of Eq. (1) at an iteration point $x_k$ is

2 $$\begin{aligned} F(x_k)+J(x_k)s=0, \end{aligned}$$

where $s=x-x_k$ and $J(x_k)$ is the Jacobian matrix of *F*(*x*) at $x_k$. For notation purposes, let $F_k=F(x_k)$ and $J_k=J(x_k)$. If $m=n$ and $J(x_k)$ is nonsingular, then the linear approximation (2) gives the Newton–Raphson iteration

3 $$\begin{aligned} x_{k+1}=x_k-J^{-1}_k F_k. \end{aligned}$$

In 1669, Newton first used the Newton iteration (2) to solve a cubic equation. In 1690 Raphson first employed the formula (3) to solve a general cubic equations. Then Fourier (1890), Cauchy (1829), and Fine (1916) established the convergence theorem of Newton’s method for different cases. In 1948, Kantorovich (1948) established the convergence theorem referred to the Newton–Kantorovich theorem. This theorem is the main tool for proving the convergence of various Newton-type methods.

There are various Newton-Type methods for solving nonlinear equations. Dembo et al. (1982) proposed an inexact Newton method. This method approximately solves the linear equation (2). Another most efficient approach is approximating the Jacobian or inverse of the Jacobian in some way. In this way, the approximation of the Jacobian satisfies the secant equation

4 $$\begin{aligned} B_ks_{k-1}=F(x_k)-F(x_{k-1}), \end{aligned}$$

where $B_k$ is an approximation for the Jacobian and $s_{k-1} = x_k - x_{k-1}$. For this kind of method, the secant equation (4) plays a vital role; therefore a wide variety of methods that satisfy the secant equation have been designed (Dennis and Schnabel 1993; Kelley 2003). Qi and Sun (1993) extended Newton’s method for solving a nonlinear equation of several variables to a nonsmooth case by using the generalized Jacobian instead of the derivative. This extension includes the B-derivative version of Newton’s method as a special case. In order to improve the convergence order of Newton-type methods, many higher order approaches have been proposed in past years. In particular, there is much literature focused on the nonlinear scalar function. Petković et al. (2013b) provide a survey, many of which are presented in the book (Petković et al. 2013a). For the nonlinear vector function *F*(*x*) in (1), there are still a lot of higher order methods. For instance, Grau-Sánchez et al. (2011), Noor and Waseem (2009), Homeier (2004), and Frontini and Sormani (2004) have proposed a third order method using one function value, two Jacobian matrices and two matrix inversions per iteration. In Darvishi and Barati (2007a), a third order method has been proposed with two function values, one Jacobian and one matrix inversion per iteration. Darvishi and Barati (2007b), and Sharma et al. (2013) developed a fourth order method. In pursuit of a higher order algorithm, researchers have also proposed fifth and sixth order methods in Grau-Sánchez et al. (2011). In summary, these higher order methods need more function values, Jacobians or matrix inversions per iteration.

In this paper, we are interested in a Newton-type method with high computational efficiency for solving the system of nonlinear equations (1). Motivated by the approach in Sui et al. (2014), we provide a new rational model $R:{\mathbb {R}}^n \rightarrow {\mathbb {R}}^m$. Although our approximation function is similar to the real valued function RALND studied in Sui et al. (2014), the proposed function is different from the RALND function. Based on this model, we linearize the nonlinear function *F*(*x*) and obtain a linear equation that is different from the first order Taylor polynomial. We then propose an improved Newton’s algorithm to solve nonlinear equations (1). In the new algorithm, in order to reflect more curvature information of nonlinear functions, the Jacobian matrix is updated by rank one matrix in each iteration. This method possesses high computational efficiency , and therefore does not increase calculation of function value, Jacobian or inverse Jacobian. Applying Newton’s method’s validation criteria, we prove that the algorithm is well-defined and the convergence rate is quadratic under some suitable conditions. The preliminary numerical experiment results and comparison are reported, showing the effectiveness of the algorithm.

This paper is organized as follows. We give a new rational approximation and improved Newton’s method in the next section. In section “Convergence analysis”, converge analysis is discussed and some numerical experiment results are reported in section “Numerical experiments”. The last section is a brief conclusion.

## Rational approximation and improved Newton’s method

Based on the information of the last two points, Sui proposed a RALND function (Sui et al. 2014) $r: {\mathbb {R}}^n \rightarrow {\mathbb {R}}$ with linear numerator and denominator that is defined by

5 $$\begin{aligned} r({x})=a_{0}+\frac{a^{\mathrm{T}}_k(x - x_{k})}{1+ b^{\mathrm{T}}_{k}(x -x_{k})}, \end{aligned}$$

where $a_k, b_k \in {\mathbb {R}}^n$ are the undetermined coefficient vectors and $x_{k}\in {\mathbb {R}}^n$ is the current point. Let

$$\begin{aligned} c_{0}=\nabla ^{\mathrm {T}} f(x_{k-1})(x_{k-1}-x_k), \quad c_{1}=\nabla ^{\mathrm {T}} f(x_k)(x_{k-1}-x_k). \end{aligned}$$

Under the following interpolation conditions

$$\begin{aligned} r(x_k)=f(x_k),\nabla r(x_k)=\nabla f(x_k), \quad \nabla r(x_{k-1})=\nabla f(x_{k-1}), \end{aligned}$$

we obtain the RALND function

6 $$\begin{aligned} r(x)=f(x_k)+\frac{\nabla ^{\mathrm {T}} f(x_k)(x-x_k)}{1+\frac{1}{c_{0}} \left(\sqrt{\frac{c_{0}}{c_{1}}}\nabla ^{\mathrm {T}} f(x_k)-\nabla ^{\mathrm {T}}f(x_{k-1})\right)(x-x_k)}, \end{aligned}$$

where $x_k\in {\mathbb {R}}^n$, $x_{k-1}\in {\mathbb {R}}^n$ are the current point and the preceding point. The RALND function has many good properties (Sui et al. 2014). For example, it is monotone with any direction and has more curvature information of the nonlinear function *F*(*x*) than the linear approximation model. These properties may be able to reduce the number of iterations when using an iteration method that was constructed by RALND to solve (1). Although the RALND function possesses some nice properties, the function $r:{\mathbb {R}}^{n}\rightarrow {\mathbb {R}}$ defined by (6) is a real valued function with each function having a different vector $b_k$. This make it more complex for nonlinear equations.

Next, we employ the RALND function with the same horizon vector $b_k$ for all nonlinear functions $F_i(x),i=1,\ldots ,n$ at $x_k$, and approximate the nonlinear equations (1) by

7 $$\begin{aligned} F(x_{k}+s)\approx R(x_{k}+s)= F_{k}+\frac{J_{k} s}{1+b^{\mathrm {T}}_{k}s}=0. \end{aligned}$$

When $b_k=0$, the rational function (7) reduces to the linear expansion (2). There is a well-known analogy between the rational function (7) and RALND (6), but the function (7) is different from (6). For the RALND function (6), each function $F_i(x),i=1,\ldots ,m$ has a different vector $b^{(k)}_i,i=1,\ldots ,m$ at current iteration point $x_k$, but the new approximation function (7) has the same vector $b_k$ for all functions $F_i(x),i=1,\ldots ,m$ at the same iteration point $x_k$. This is the main difference between the two functions (7) and (6). Because of this difference, the function (7) is more suitable for nonlinear equations.

Similar to the linearization approach in (2), from approximate equations (7) we can obtain a new iterative formula

8 $$\begin{aligned} (J_{k} + F_{k} b^{\mathrm {T}}_{k}) s_k=-F_{k}. \end{aligned}$$

If the matrix $J_k + F_k b^{T}_k$ is invertible, it follows that

9 $$\begin{aligned} x_{k+1}=x_k -(J_k + F_k b^{T}_k)^{-1}F_k. \end{aligned}$$

when $b_k=0$, the iterative scheme (8) and (9) reduce to the linear equations (2) and Newton–Raphson iteration (3), respectively.

Moreover, Davidon proposed the conic model (Davidon 1980; Sorensen 1980) and many researchers have studied the conic model and collinear scaling algorithms (Ariyawansa 1990; Ariyawansa and Lau 1992; Deng and Li 1995; Gourgeon and Nocedal 1985). Near the current iteration point $x_k$, the conic function *c*(*x*) is defined by

10 $$\begin{aligned} f(x)\approx c_k(x)= f(x_k)+ \frac{\nabla f(x_k)^{\mathrm {T}}s}{1+b^\mathrm {T}_k s}+\frac{s^\mathrm {T}B_k s}{2\left( 1+b^\mathrm {T}_k s\right) ^2}. \end{aligned}$$

In the conic model (10), the horizon vector $b_k$ is a parameter. This parameter gives the conic model more freedom. Many researchers have given more attention to $b_k$. As a result, some methods of choosing the horizon vector have been developed (Davidon 1980; Deng and Li 1995; Sheng 1995). Interestingly, the function (7) is the first two terms of conic model (10). In what follows we use these methods to determine the vector $b_k$ in (7).

After a step from $x_{k-1}$ to $x_{k}$, we update $b_{k-1}$ to $b_{k}$ by requiring the following extra interpolation condition

11 $$\begin{aligned} R(x_{k-1})=F(x_{k-1}). \end{aligned}$$

This causes the search direction in (9) to depend on the Jacobian of the current point and the function values of the preceding point as well as the current point. In Newton’s method the search direction is determined by the Jacobian and function value of the current point. Compared with Newton’s method, more flexibility and more accurate approximation of the nonlinear function may be expected for the rational model (7).

From (11) we have

12 $$\begin{aligned} F_{k-1}=F_{k}-\frac{J_{k}s_{k-1}}{1-b^\mathrm {T}_{k}s_{k-1}}, \end{aligned}$$

where $$s_{k-1}=x_{k}-x_{k-1}$$. Let

13 $$\begin{aligned} \beta _{k}= 1-b^\mathrm {T}_{k} s_{k-1}, \end{aligned}$$

14 $$\begin{aligned} y_{k-1}= F_{k}-F_{k-1}. \end{aligned}$$

Considering (12), we get

$$\begin{aligned} \beta _{k} y_{k-1} =J_{k}s_{k-1}, \end{aligned}$$

thus

15 $$\begin{aligned} \beta _{k}=\frac{y^\mathrm {T}_{k-1} J_{k}s_{k-1}}{y^\mathrm {T}_{k-1} y_{k-1}}. \end{aligned}$$

Note that

16 $$\begin{aligned} b_k = \frac{(1-\beta _k)a_k}{a^\mathrm {T}_k s_{k-1}} \end{aligned}$$

for any $a_k\in {\mathbb {R}}^n$ with $a^\mathrm {T}_{k}s_{k-1}\ne 0$, will satisfy (13). Considering the special choice $a_k = s_{k-1}$, we have

17 $$\begin{aligned} b_{k}=\frac{(1-\beta _{k})s_{k-1}}{s^\mathrm {T}_{k-1} s_{k-1}}=\frac{y^\mathrm {T}_{k-1}(y_{k-1} - J_{k}s_{k-1})s^\mathrm {T}_{k-1}}{\left( y^\mathrm {T}_{k-1} y_{k-1}\right) \left( s^\mathrm {T}_{k-1} s_{k-1}\right) }. \end{aligned}$$

Analogously, we can consider another method (Sheng 1995) for constructing horizon vectors. Using (17) and (15), we see that

18 $$\begin{aligned} F_{k} b^\mathrm {T}_{k}= \frac{y^\mathrm {T}_{k-1}(y_{k-1} -J_{k}s_{k-1})}{y^\mathrm {T}_{k-1}y_{k-1}}\frac{F_{k}s^\mathrm {T}_{k-1}}{s^\mathrm {T}_{k-1} s_{k-1}}. \end{aligned}$$

Next, we give the improved Newton’s method for system of nonlinear equations.
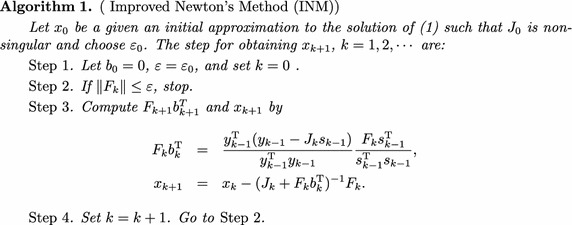


There are two differences between Algorithm 1 and Newton’s method. First, INM uses the rank one technique to revise the Jacobian in every iteration. Second, INM utilises the function values of the previous iteration point.

For the one dimensional nonlinear equation $f(x)=0$, where $f:{\mathbb {R}}\rightarrow {\mathbb {R}}$ is continuously differentiable on $D \subset {\mathbb { R}}$, the nonlinear function of $f(x)$ is approximated by

$$\begin{aligned} f(x)\approx f(x_k)+\frac{f'(x_k)(x-x_k)}{1+b_k(x-x_k)}. \end{aligned}$$

Then, we have

19 $$\begin{aligned} x_{k+1}=x_k - \frac{f(x_k)}{f'(x_k)+f(x_k)b_k}. \end{aligned}$$

We also use the interpolation method to determined the parameter $b_k$ by

20 $$\begin{aligned} b_k=\frac{f(x_k)-f(x_{k-1})-f'(x_{k})(x_k-x_{k-1})}{(f(x_{k})-f(x_{k-1}))(x_k-x_{k-1})}. \end{aligned}$$

Then (20) together with (19) gives the following iteration scheme

21 $$\begin{aligned} x_{k+1}=x_k-\frac{f(x_k)}{\frac{f'(x_{k})f(x_{k-1})}{f(x_{k-1})-f(x_{k})}+\frac{f(x_{k})}{x_k-x_{k-1}}}. \end{aligned}$$

This is a new modified Newton formula.

## Convergence analysis

In this section, we prove the local quadratic convergence of Algorithm 1 for system of nonlinear equations. The techniques of the proof are similar to Newton’s method for nonlinear equations. In the rest of this paper, we make the following assumptions:

### **Assumption 1**

(i) $J(x^*)$ is nonsingular and there exist a constant $\mu > 0$, such that $\Vert J(x^*)\Vert \le \mu$.
(ii) The function *F* is continuously differentiable in the open convex set $D\subset {\mathbb {R}}^n$, and there exists a constant $\gamma >0$, such that for all $x,y\in D$
  $$\begin{aligned} \Vert J(x) - J(y)\Vert \le \gamma \Vert x-y\Vert . \end{aligned}$$

For proving the convergence theorem we need the following Lemmas.

### **Lemma 1**

*Let *$F:{\mathbb {R}}^n\rightarrow {\mathbb {R}}^m$*satisfy the* (ii) *of Assumption*1. *Then for any*$x+s\in D$,

22 $$\begin{aligned} \Vert F(x+s)-F(x)-J(x)s\Vert \le \frac{\gamma }{2}\Vert s\Vert ^2. \end{aligned}$$

### *Proof*

Please refer to Lemma 4.1.12 in Dennis and Schnabel (1993). $\square$

### **Lemma 2**

*Let F*, *Jsatisfy the conditions of Lemma*1, *and assume that*$J(x^*)$*exists. Then there exist*$\varepsilon >0$*and*$0<m<M$, *such that*

23 $$\begin{aligned} m\Vert v-u\Vert \le \Vert F(v)-F(u)\Vert \le M\Vert v-u\Vert , \end{aligned}$$

*for all*$v,u\in D$*for which*$\max \{\Vert v-x^*\Vert ,\Vert u-x^*\Vert \}\le \varepsilon$.

### *Proof*

Please refer to Lemma 4.1.16 in Dennis and Schnabel (1993). $\square$

With the help of the preceding two lemmas we can prove the following Theorem of convergence. We denote the epsilon neighborhood of $x_*$ by $N(x_*,\varepsilon )$, i.e.,

$$\begin{aligned} N(x_*,\varepsilon ) = \{x, \ \Vert x - x_*\Vert \le \varepsilon , \forall x \in {\mathbb {R}} \}. \end{aligned}$$

### **Theorem 1**

*Let*$F:{\mathbb {R}}^n\rightarrow {\mathbb {R}}^n$*satisfy Assumption*1*and suppose that there exist*$x_*\in {\mathbb {R}}^n$, $m>0$*and*$r > 0$, *such that*$N(x_*,r)\subset D$, $F(x_*)=0$. *Then there exist*$\varepsilon >0$*such that for all*$x_0\in N(x_*,\varepsilon )$*the sequence*$\{x_2,x_3,\cdots \}$*generated by Algorithm *1 *is well defined, converges to*$x_*$, *and obeys*

24 $$\begin{aligned} \Vert x_{k+1}-x_*\Vert \le \frac{\mu \gamma (m+\gamma )}{m}\Vert x_{k}-x_*\Vert ^2, \quad k=1,2,\ldots \end{aligned}$$

### *Proof*

Since $b_0=0$, we obtain the following inequality from the proof of Newton’s method (Dennis and Schnabel 1993),

25 $$\begin{aligned} \Vert x_1-x_*\Vert \le \frac{1}{2}\Vert x_0-x_*\Vert . \end{aligned}$$

Let

$$\begin{aligned} \varepsilon = \min \left\{ r, \frac{2m}{\mu \gamma (2m+\gamma )}\right\} . \end{aligned}$$

By a routine computation,

$$\begin{aligned} \left\| J(x_*)^{-1}\left[ \left( J_1+F_1b^T_1 \right) -J(x_*)\right] \right\| & \le \Vert J(x_*)^{-1}\Vert \left( \left\| J_1-J(x_*)\right\| +\left\| F_1b^T_1 \right\| \right) \\ & \le \mu \left( \left\| J_1 - J(x_*)\right\| +\left\| F_1 b^T_1\right\| \right) \\ & \le \mu \left( \gamma \left\| x_1-x_*\right\| +\left\| F_1 b^T_1 \right\| \right) . \end{aligned}$$

Considering the second term of the above expression, it follows from (22) and (23) that

$$\begin{aligned} \left\| F_1 b^T_1 \right\| & = \Vert F_{1}\Vert \Vert b_1\Vert \le \Vert F_{1}\Vert \left\| \frac{y^\mathrm {T}_0(F_1- F_0 -J_{1}s_0)}{y^\mathrm {T}_0 y_0}\right\| \frac{1}{\Vert s_0\Vert } \\ & \le \Vert F_{1}\Vert \frac{\Vert (F_1- F_0 - J_{1}s_0)\Vert }{\Vert y_0\Vert \Vert s_0\Vert } \le \Vert F_{1}\Vert \frac{\gamma \Vert s_0\Vert }{2\Vert y_0\Vert } \\ & \le \Vert F_{1}\Vert \frac{\gamma }{2m} = \frac{\gamma }{2m} \Vert F_{1}-F_*\Vert \\ & \le \frac{\gamma ^2}{2m} \Vert x_1-x_*\Vert . \end{aligned}$$

Then,

26 $$\begin{aligned} \left\| J(x_*)^{-1}\left[ \left( J_1+F_1b^T_1 \right) -J(x_*)\right] \right\| & \le \mu \gamma \left( 1 + \frac{\gamma }{2m}\right) \Vert x_1-x_*\Vert \nonumber \\ & \le \frac{\mu \gamma }{2} \left( 1 + \frac{\gamma }{2m}\right) \Vert x_0-x_*\Vert \nonumber \\ & \le \frac{\mu \gamma }{2}\left( 1 + \frac{\gamma }{2m}\right) \varepsilon \le \frac{1}{2}. \end{aligned}$$

Therefore, by the perturbation theorem, $J_1+F_1b^T_1$ is nonsingular and

27 $$\begin{aligned} \left\| \left( J_1+F_1b^T_1\right) ^{-1}\right\| & = \frac{ \left\| J(x_*)^{-1}\right\| }{1- \left\| J(x_*)^{-1}\left[ \left( J_1+F_1b^T_1 \right) -J_*)\right] \right\| } \nonumber \\ & \le 2 \Vert J(x_*)^{-1}\Vert \nonumber \\ & \le 2\mu . \end{aligned}$$

Thus $x_2$ is well defined. From our method, we get

$$\begin{aligned} x_2 - x_* & = x_1 - x_* -\left( J_1 + F_1b^\mathrm {T}_1\right) ^{-1}F_1 \\ & = x_1 - x_* -\left( J_1 + F_1b^\mathrm {T}_1\right) ^{-1}(F_1-F_*) \\ & = \left( J_1 + F_1b^\mathrm {T}_1\right) ^{-1}\left[ F_* -F_1 - \left( J_1 + F_1b^\mathrm {T}_1\right) (x_* - x_1)\right] . \end{aligned}$$

Furthermore,

$$\begin{aligned} \Vert x_2 - x_*\Vert & \le \left\| \left( J_1 + F_1b^\mathrm {T}_1\right) ^{-1}\right\| \left\| F_* -F_1 - \left( J_1 + F_1b^\mathrm {T}_1\right) (x_* - x_1)\right\| \\ & \le 2\mu \left\| F_* -F_1 - \left( J_1 + F_1b^\mathrm {T}_1\right) (x_* - x_1)\right\| \\ & \le 2\mu \left( \frac{\gamma }{2}\Vert (x_* - x_1)\Vert ^2+\left\| F_1b^\mathrm {T}_1(x_* - x_1)\right\| \right) \\ & \le 2\mu \left( \frac{\gamma }{2}\Vert (x_* - x_1)\Vert ^2 + \frac{\gamma ^2}{2m}\Vert x_*-x_1\Vert ^2\right) \\ & = \frac{\mu \gamma (m+\gamma )}{m}\Vert x_* - x_1\Vert ^2. \end{aligned}$$

This proves (24). Taking (25) into consideration leads to

$$\begin{aligned} \Vert x_2 - x_*\Vert & \le \frac{\mu \gamma (m+\gamma )}{4m}\Vert x_0-x_*\Vert ^2 \\ & \le \frac{\mu \gamma (m+\gamma )}{4m}\varepsilon \Vert x_0-x_*\Vert \\ & < \frac{1}{2} \Vert x_0-x_*\Vert . \end{aligned}$$

Then $x_2\in N(x_*,r)$ and completes the case $k=1$. The proof of the induction step proceeds identically. $\square$

## Numerical experiments

This section is devoted to the numerical results. First, we show the numerical comparison between Algorithm 1, Newton’s method and a third order Newton’s method for finding a root of real function. This provides the numerical evidence that Algorithm 1 is better then Newton’s method. Secondly, we demonstrate the performance of Algorithm 1 for solving system of nonlinear equations. Algorithm 1 has been applied to some popular test problems and compared with Newton’s method and a third order method. All codes were written in Mathematica10.0 and run on a PC with an Intel i7 3.6GHz CPU processor, 4GB memory and 64-bit Windows 7 operating system.

### Finding roots of real function

In this subsection we demonstrate the performance of our improved Newton’s method for finding the root of real functions $f:{\mathbb {R}}\rightarrow {\mathbb {R}}$. In other words, we show the efficiency of the new iteration formula (21) in solving a root of the nonlinear equation. Specifically, we chose ten particular nonlinear equations from the literature (Thukral 2016) which are listed in Table 1.

**Table 1:** Test equations and range of initial point

Equation	Range of initial
$f_1(x) = \exp (x)\sin (x)+\ln (1+x^2)=0$	$x_0\in [-0.1,1]$
$f_2(x) = \exp (x)\sin (x)+\cos (x)\ln (1+x)=0$	$x_0\in [-1,1]$
$f_3(x) = \exp (\sin (x))-x/5 -1 =0$	$x_0\in [-0.5,1]$
$f_4(x) = (x+1)\exp (\sin (x))-x^2\exp (\cos (x))=0$	$x_0\in [-1.5,1]$
$f_5(x) = \sin (x)+\cos (x)+\tan (x)-1=0$	$x_0\in [-1,1]$
$f_6(x) = \exp (-x)-\cos (x)=0$	$x_0\in [-1,0.5]$
$f_7(x) = \ln (1+x^2)+\exp (x^2-3x)\sin (x)=0$	$x_0\in [-0.2,1]$
$f_8(x) = x^3+\ln (1+x)=0$	$x_0\in [-0.5,1]$
$f_9(x) = \sin (x)-x/3=0$	$x_0\in [-0.5,1]$
$f_5(x) = (x-10)^6-10^6=0$	$x_0\in [-1,1]$

In our tests, the stopping criteria used are $\Vert F(x_k)\Vert <10^{-6}$ or the number of iterations exceeds 100. We compute these 10 problems by using the iteration formula (21), Newton’s Method and a third order Newton’s Method introduced in Darvishi and Barati (2007a). In our experiments, the initial point for each problem is randomly generated ten times in the range of the initial point, and the average numerical results are listed in Table 2, where

INM: denotes the iteration formula (21),
NM: denotes Newton’s method,
3NM: denotes the third order Newton’s method (Darvishi and Barati 2007a),
It: denotes the average number of iterations,
Re: denotes the average value of $|f(x_k)|$ when the iteration stop,
Fa: denotes the number of failures in solving equations.

From Table 2, in terms of the number of iterations, the efficiency of the improved Newton formula (21) is better than Newton’s method, but not as good as the third order method.

**Table 2:** Numerical experiment results of INM, NM and 3NM

Equation	INM			NM			3NM		
	It	Re	Fa	It	Re	Fa	It	Re	Fa
$f_1$	3.3	9.1830E−8	0	4.5	7.6091E−8	0	3.2	1.1458E−7	0
$f_2$	3.0	2.5350E−9	0	4.0	9.4571E−10	0	3.0	1.3982E−13	0
$f_3$	2.8	1.5251E−8	0	3.1	1.7424E−7	0	2.3	1.9551E−7	0
$f_4$	3.1	4.0420E−8	0	3.5	2.1129E−9	0	2.7	4.9111E−8	0
$f_5$	3.0	1.1038E−8	0	3.6	5.9695E−9	0	2.6	4.9111E−8	0
$f_6$	3.7	2.4752E−8	0	4.3	1.1354E−7	0	2.9	9.2102E−8	0
$f_7$	3.3	1.400E−8	0	3.9	1.2565E−7	0	2.6	4.6376E−8	0
$f_8$	3.2	1.3959E−7	0	3.7	1.2625E−9	0	2.6	2.2066E−9	0
$f_9$	3.7	2.7669E−9	0	5.8	5.1190E−8	0	2.6	4.3207E−8	0
$f_{10}$	3.2	5.4249E−9	0	4.3	1.2257E−7	0	2.9	1.3057E−7	0

To compare the performance of the iteration formula (21), Newton’s method and the third order method (Darvishi and Barati 2007a), we consider the performance profile introduced in Dolan and More (2002) as a means. We assume that there are $n_s$ solvers and $n_p$ test problems from the test set $\mathcal {P}$ which is chosen from Table 1. The initial point is selected randomly from the range of the initial point. We are interested in using the iteration number as a measure of performance for the iteration formula (21), NM and 3NM. For each problem *p* and solver *s*, let

$$\begin{aligned} f_{p,s} = \hbox {iteration number required to solve problem } p\hbox { by solver }s. \end{aligned}$$

We employ the performance ratio

$$\begin{aligned} r_{p,s} := \frac{f_{p,s}}{\min \{f_{p,s}:s\in \mathcal {S}\}}, \end{aligned}$$

where $\mathcal {S}$ is the three solvers set. We assume that a parameter $r_{M} \ge r_{p,s}$ is chosen for all *p*, *s*, and $r_{p,s} = r_{M}$ if and only if solver *s* does not solve problem *p*. In order to obtain an overall assessment for each solver, we define

$$\begin{aligned} \rho _s(\tau ) := \frac{1}{n_p}\mathrm {size}\{p \in \mathcal {P}:r_{p,s}\le \tau \}, \end{aligned}$$

which is called the performance profile of the number of iterations for solver *s*. Then, $\rho _s(\tau )$ is the probability for solver $s\in \mathcal {S}$ that a performance ratio $f_{p,s}$ is within a factor $\tau \in {\mathbb {R}}$ of the best possible ratio.

**Fig. 1:**
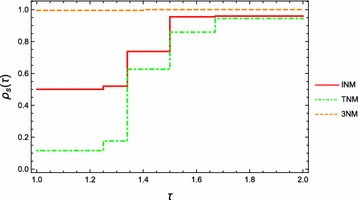
Performance profile of iteration numbers of INM, NM and 3NM

Figure 1 shows the performance profile of iteration numbers in the range of $\tau \in [1,2]$ for three solvers on 200 test problem which were selected from Table 1 with random initial points. From this figure, we see that the numerical performance of solver INM is between 3NM and NM. In summary, from the viewpoint of iteration numbers, we conclude that

$$\begin{aligned} \text {3NM}> \text {INM} > \text {NM}, \end{aligned}$$

where “>” means “better performance”.

### Solving system of nonlinear equations

In this subsection we show the numerical efficiency of Algorithm 1 for solving system of nonlinear equations. Listed in Table 3 are the 12 multivariable test problems that were chosen from the test problems set (Dennis and Schnabel 1993; Moré et al. 1981; Andrei 2008). The starting points for each problem are the standard starting points. Illustrative examples further demonstrate the superiority of our proposed algorithm. The numerical results are listed in Table 4, where

INM: denotes Algorithm 1,
NM: denotes Newton’s method,
3NM: denotes the third order Newton method (Darvishi and Barati 2007a),
Dim: denotes the size of problem,
It: denotes the number of iterations,
Ti: denotes the value of the CPU time in seconds,
–: denotes that the number of iterations exceeded 100.

It is observed from Table 4 that in terms of the number of iterations and computation time, the efficiency of Algorithm 1 is better than Newton’s method for most of the testing problems, and the efficiency of Algorithm 1 is close to the third order convergence method 3NM (Darvishi and Barati 2007a).

**Table 3:** Test problems

Function	Name	Function	Name
F0	Rosenbrock	F1	Powell badly scaled
F2	Freudenstein and Roth	F3	Powell singular
F4	Trigonometric	F5	Trigonometric exponential
F6	Trigexp	F7	Broyden tridiagonal
F8	Extend Power singular	F9	Discrete boundary
F10	Discrete integral equation	F11	Broyden banded

**Table 4:** Numerical experiment results of INM, NM and 3NM

Pr	Dim	INM		NM		3NM		Pr	Dim	INM		NM		3NM	
		It	Ti	It	Ti	It	Ti			It	Ti	It	Ti	It	Ti
F0	2	2	0.1E−8	2	0.1E−8	1	0.1E−8	F1	2	7	0.001	11	0.001	8	0.1E−8
F2	2	27	0.005	42	0.005	–	–	F3	2	9	0.001	11	0.001	8	0.001
F4	10	6	0.008	7	0.007	–	–	F5	10	8	0.007	10	0.007	19	0.021
F4	50	5	0.349	9	0.641	–	–	F5	50	8	0.121	10	0.187	19	2.589
F4	100	5	2.193	9	3.811	–	–	F5	100	8	0.858	10	0.998	19	3.661
F4	500	6	0.008	7	0.007	–	–	F5	500	8	61.71	10	75.82	19	141.9
F6	10	5	0.015	5	0.001	4	0.015	F7	10	6	0.1E−8	7	0.1E−8	–	–
F6	50	5	0.140	5	0.125	4	0.125	F7	50	8	0.078	10	0.094	–	–
F6	100	5	0.702	5	0.764	4	0.733	F7	100	9	0.533	11	0.633	–	–
F6	500	5	37.39	5	35.820	4	32.00	F7	500	11	59.18	14	75.53	–	–
F8	8	11	0.003	13	0.003	9	0.004	F9	10	2	0.002	2	0.002	2	0.002
F8	60	11	0.212	13	0.239	10	0.209	F9	50	2	0.020	2	0.020	1	0.015
F8	100	11	0.854	13	0.953	10	0.837	F9	100	2	0.140	2	0.136	1	0.082
F8	500	12	71.61	14	83.23	10	62.05	F9	500	1	6.515	1	6.038	1	5.966
F10	10	2	0.004	2	0.004	2	0.005	F11	10	5	0.005	5	0.004	4	0.005
F10	50	2	0.291	2	0.278	2	0.005	F11	50	5	0.114	5	0.123	4	0.143
F10	100	2	2.183	3	3.216	2	3.087	F11	100	5	0.567	5	0.623	4	0.630
F10	500	2	284.2	2	355.3	2	352.8	F11	500	5	33.84	5	32.76	4	29.70

**Fig. 2:**
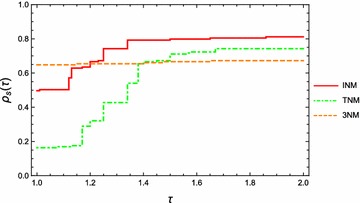
Performance profile of INM, NM and 3NM

The above experiments were conducted on the standard initial point. We then also need to test the three methods for test problems (Table 3) at random starting points. In particular, starting points for each problem are randomly chosen 10 times from a box surrounding the standard starting points. In order to obtain an overall assessment for the three methods, we are also interested in using the number of iterations as a performance measure for Algorithm 1, Newton’s method and the third order method (Darvishi and Barati 2007a). The performance plot based on iteration number is presented in Fig. 2. From this figure, we can see that Algorithm 1 has the best performance for $\tau > 1.3$. Again, from the viewpoint of large test problems with a perturbed initial point, we conclude that Algorithm 1 is better than Newton’s method or the third order method (Darvishi and Barati 2007a).

## Conclusion

In this paper, we present an improved Newton’s method for system of nonlinear equations by re-use of the previous iteration information. In the novel method, the function value of the previous iteration point was utilized for correcting the Newton direction. The proposed new method also has the quadratic convergence property. From the numerical results obtained for a set of standard test problems, it appears that the rank one revised implementation scheme described, where the Jacobian matrix is updated by a rank one matrix, may allow considerable computational savings for iteration number and computing time. Moreover, two kinds of numerical comparisons are presented in this paper. The first one is the numerical comparison between the new Newton formula, Newton’s method and a third order Newton method for finding roots of scalar functions. From this comparison we see that the proposed algorithm is efficient for one dimensional real function. The second comparison is for multivariate vector equations. From this comparison we see that the numerical performance of the proposed algorithm in the case of multidimensional is better than the one-dimensional case. This is a very interesting discovery which may be helpful in other contexts.

## References

[CR1] Andrei N (2008). An unconstrained optimization test functions collection. Adv Model Optim.

[CR2] Ariyawansa KA (1990). Deriving collinear scaling algorithms as extensions of quasi-Newton methods and the local convergence of DFP-and BFGS-related collinear scaling algorithms. Math Program.

[CR3] Ariyawansa KA, Lau DTM (1992). Local and Q-superlinear convergence of a class of collinear scaling algorithms that extends quasi-newton methods with broyden’s bounded class of updates. Optimization.

[CR4] Cauchy AL (1829) *Sur la détermination approximative des racines d’une équation algébrique ou transcendante*. In: Lecons sur le calcul differentiel, Buré fréres, Paris, pp 575–600

[CR5] Darvishi MT, Barati A (2007). A third-order Newton-type method to solve system of nonlinear equations. Appl Math Comput.

[CR6] Darvishi MT, Barati A (2007). Afourth-order method from quadrature formulae to solve systems of nonlinear equations. Appl Math Comput.

[CR7] Davidon WC (1980). Conic approximation and collinear Horizontal for optimizer. SIAM J Numer Anal.

[CR8] Dembo RS, Eisenstat SC, Steihaug T (1982). Inexact newton methods. SIAM J Numer Anal.

[CR9] Deng NY, Li ZF (1995). Some global convergence properties of a conic-variable metric algorithm for minimization with inexact line searches. Optim Methods Softw.

[CR10] Dennis JE, Schnabel RB (1993). Numerical methods for unconstrained optimization and nonlinear equations.

[CR11] Dolan ED, More JJ (2002). Benchmarking optimization software with performance profiles. Math Program.

[CR12] Fine HB (1916). On Newton’s method of approximation. Proc Natl Acad Sci USA.

[CR13] Fourier JBJ (1890) *Question d’analyse algébrique*. In: Oeuvres complétes(2), Gauthier-Villars, Paris, pp 243–253

[CR14] Frontini M, Sormani E (2004). Third-order methods from quadrature formulae for solving systems of nonlinear equations. Appl Math Comput.

[CR15] Gourgeon H, Nocedal J (1985). A conic algorithm for optimization. SIAM J Sci Stat Comput.

[CR16] Grau-Sánchez M, Grau A, Noguera M (2011). On the computational efficiency index and some iterative methods for solving systems of nonlinear equations. J Comput Appl Math.

[CR17] Homeier HHH (2004). Amodified Newton method with cubic convergence: the multivariable case. J Comput Appl Math.

[CR18] Kantorovich LL (1948). On Newton’s method for functional equations. Dokl Akad Nauk SSSR.

[CR19] Kelley CT (2003). Solving nonlinear equations with Newton’s method.

[CR20] Moré JJ, Garbow BS, Hillstrom KE (1981). Testing unconstrained optimization software. ACM Trans Math Softw.

[CR21] Noor MA, Waseem M (2009). Some iterative methods for solving a system of nonlinear equations. Comput Math Appl.

[CR22] Ortega JM, Rheinboldt WC (1970). Iterative solution of nonlinear equations in several variables.

[CR23] Petković MS, Neta B, Petković LD, Džunić J (2013). Multipoint methods for solving nonlinear equations.

[CR24] Petković MS, Neta B, Petković LD, Džunić J (2013). Multipoint methods for solving nonlinear equations: a survy. Appl Math Comput.

[CR25] Qi L, Sun J (1993). A nonsmooth version of Newton’s method. Math Program.

[CR26] Sharma JR, Guha RK, Sharma R (2013). An efficient fourth order weighted-Newton method for systems of nonlinear equations. Numer Algorithms.

[CR27] Sheng S (1995). Interpolation by conic model for unconstrained optimization. Computing.

[CR28] Sorensen DC (1980). The q-superlinear convergence of a collinear scaling algorithm for unconstrained optimization. SIAM J Numer Anal.

[CR29] Sui Y, Saheya, Chen, G (2014) An improvement for the rational approximation RALND at accumulated two-point information. Math Numer Sinica 36(1):51–64

[CR30] Thukral R (2016). New modification of Newton method with third-order convergence for solving nonlinear equations of type $$f(0)=0$$. Am J Comput Appl Math.

[CR31] Traub JF (1964). Iterative method for the solution of equations.

